# Accuracy of heart rate apps varies

**Published:** 2017

## Accuracy of heart rate apps varies

Consumers are being warned about the accuracy of heart rate apps after a study found huge variability between commercially available apps, even those using the same technology. The research is published in the European Journal of Preventive Cardiology.^1^

‘Heart rate apps come installed on many smartphones and once people see them it is human nature to use them and compare their results with others’, said last author Dr Christophe Wyss, a cardiologist at Heart Clinic Zurich, Switzerland. ‘The problem is that there is no law requiring validation of these apps and therefore no way for consumers to know if the results are accurate.’

This study tested the accuracy of four commercially available heart rate apps (randomly selected) using two phones, the iPhone 4 and iPhone 5. Some apps use contact photoplethysmography (touching fingertip to the phone’s built-in camera) while other apps use non-contact photoplethysmography (camera is held in front of the face).

Accuracy was assessed by comparing the results with the clinical gold-standard measurements. These are the electrocardiogram (ECG), which measures the electrical activity of the heart using leads on the chest, and fingertip pulse oximetry, which uses photoplethysmography.

The study included 108 patients who had their heart rate measured by ECG, pulse oximetry, and each app using each phone.

The researchers found substantial differences in accuracy between the four apps. In some apps there were differences of more than 20 beats per minute compared to ECG in over 20% of the measurements. The non-contact apps performed less well than the contact apps, particularly at higher heart rates and lower body temperatures. The non-contact apps had a tendency to overestimate higher heart rates.

Dr Wyss said: ‘While it’s easy to use the non-contact apps – you just look at your smartphone camera and it gives your heart rate – the number it gives is not as accurate as when you have contact with your smartphone by putting your fingertip on the camera.’

But the performance of the two contact apps was also different. One app measured heart rate with comparable accuracy to pulse oximetry but the other app did not give the correct measurement. ‘The one contact app was excellent, performing almost like a medically approved pulse oximeter device, but the other app was not accurate even though they use the same technology’, said Dr Wyss.

The researchers tried to find the reason for the difference in performance between the two contact apps, but they found that the variation could not be explained by camera technology (iPhone 4 versus iPhone 5), age, body temperature, or heart rate itself.

‘The difference in performance between the contact apps is probably down to the algorithm the app uses to calculate heart rate, which is commercially confidential’, said Dr Wyss. ‘It means that just because the underlying technology works in one app doesn’t mean it works in another one and we can’t assume that all contact heart rate apps are accurate.’

Dr Wyss said: ‘Before you measure your heart rate, have a specific question in mind, don’t just measure it for fun. For example, “is my heart rate too high when I feel something strange in my heart?” or “is it too low when I feel dizzy?”.’

He concluded: ‘Consumers and interpreting physicians need to be aware that the differences between apps are huge and there are no criteria to assess them. We also don’t know what happens to the heart rate data and whether it is stored somewhere, which could be an issue for data protection.’
